# A Randomised Phase 2 Trial of Intensive Induction Chemotherapy (CBOP/BEP) and Standard BEP in Poor-prognosis Germ Cell Tumours (MRC TE23, CRUK 05/014, ISRCTN 53643604)

**DOI:** 10.1016/j.eururo.2014.06.034

**Published:** 2015-03

**Authors:** Robert A. Huddart, Rhian Gabe, Fay H. Cafferty, Philip Pollock, Jeff D. White, Jonathan Shamash, Michael H. Cullen, Sally P. Stenning, for the TE23 Trial Management Group and Collaborators and the National Cancer Research Institute Testis Cancer Clinical Studies Group

**Affiliations:** a The Institute of Cancer Research and The Royal Marsden NHS Foundation Trust, Sutton, UK; b Medical Research Council Clinical Trials Unit, London, UK; c Beatson West of Scotland Cancer Centre, Glasgow, UK; d St. Bartholomew's Hospital, London, UK; e University Hospitals Birmingham NHS Foundation Trust, Birmingham, UK

**Keywords:** Metastatic germ cell tumour, Poor prognosis, Randomised trial

## Abstract

**Background:**

Standard chemotherapy for poor-prognosis metastatic nonseminoma has remained bleomycin, etoposide, and cisplatin (BEP) for many years; more effective regimens are required.

**Objective:**

To explore whether response rates with a new intensive chemotherapy regimen, CBOP/BEP (carboplatin, bleomycin, vincristine, cisplatin/BEP), versus those in concurrent patients treated with standard BEP justify a phase 3 trial.

**Design, setting, and participants:**

We conducted a phase 2 open-label randomised trial in patients with germ cell tumours of any extracranial primary site and one or more International Germ Cell Cancer Collaborative Group poor-prognosis features. Patients were randomised between 2005 and 2009 at 16 UK centres.

**Intervention:**

BEP (bleomycin 30 000 IU) was composed of four cycles over 12 wk. CBOP/BEP was composed of 2 × CBOP, 2 × BO, and 3 × BEP (bleomycin 15 000 IU).

**Outcome measurements and statistical analysis:**

Primary end point was favourable response rate (FRR) comprising complete response or partial response and normal markers. Success required the lower two-sided 90% confidence limit to exclude FRRs <60%; 44 patients on CBOP/BEP gives 90% power to achieve this if the true FRR is ≥80%. Equal numbers were randomised to BEP to benchmark contemporary response rates.

**Results and limitations:**

A total of 89 patients were randomised (43 CBOP/BEP, 46 BEP); 40 and 41, respectively, completed treatment. CBOP/BEP toxicity, largely haematologic, was high (96% vs 63% on BEP had Common Terminology Criteria for Adverse Events v.3 grade ≥3). FRRs were 74% (90% confidence interval [CI], 61–85) with CBOP/BEP, 61% with BEP (90% CI, 48–73). After a median of 58-mo follow-up, 1-yr progression-free survival (PFS) was 65% and 43%, respectively (hazard ratio: 0.59; 95% CI, 0.33–1.06); 2-yr overall survival (OS) was 67% and 61%. Overall, 3 of 14 CBOP/BEP and 2 of 18 BEP deaths were attributed to toxicity, one after an overdose of bleomycin during CBOP/BEP. The trial was not powered to compare PFS.

**Conclusions:**

The primary outcome was met, the CI for CBOP/BEP excluding FRRs <61%, but CBOP/BEP was more toxic. PFS and OS data are promising but require confirmation in an international phase 3 trial.

**Patient summary:**

In this study we tested a new, more intensive way to deliver a combination of drugs often used to treat men with testicular cancer. We found that response rates were higher but that the CBOP/BEP regimen caused more short-term toxicity. Because most patients are diagnosed when their cancer is less advanced, it took twice as long to complete the trial as expected. Although we plan to carry out a larger trial, we will need international collaboration.

**Trial registration:**

ISRCTN53643604; http://www.controlled-trials.com/ISRCTN53643604.

## Take Home Message

In patients with poor-prognosis metastatic germ cell tumours, this randomised phase 2 trial shows that CBOP/BEP chemotherapy (carboplatin, bleomycin, vincristine, cisplatin/bleomycin, etoposide, cisplatin) achieves superior response rates to standard BEP chemotherapy, although with increased toxicity; efficacy results require confirmation in a phase 3 trial.

## 1 Introduction

The management of metastatic germ cell tumours (GCTs) with platinum-based chemotherapy represents a major success story. However, a poor prognostic group can be defined that achieved cure rates <50% in an international pooled analysis [1].

Attempts to improve outcomes include use of multiagent regimens (eg, cisplatin, vincristine, methotrexate, bleomycin, actinomycin D, cyclophosphamide, etoposide [POMB/ACE] [2]; bleomycin, vincristine, cisplatin/etoposide, ifosfamide, cisplatin, and bleomycin [BOP/VIP-B] [3]); new drugs such as ifosfamide [4], and high-dose chemotherapy [5], [6], [7], but none proved superior to bleomycin, etoposide, and cisplatin (BEP) in randomised trials. Rapid proliferation [8], [9], [10], [11], [12] in GCTs [13] could contribute to treatment failure; therefore, the Royal Marsden Testicular Tumour Unit developed an intensive induction regimen (BOP/BEP) based on Wettlaufer et al. [14]. Features included weekly cisplatin for 4 wk with weekly bleomycin and vincristine for 6 wk. In weeks 2 and 4, bleomycin was administered as 5-d infusions [15] rather than bolus injections [16]. Three courses of BEP followed with bleomycin at 15 000 IU/wk. Later, carboplatin was added (weeks 2 and 4), and cisplatin was given over 2 rather than 5 d (weeks 1 and 3). The resulting carboplatin, bleomycin, vincristine, cisplatin/bleomycin, etoposide, cisplatin (CBOP/BEP) regimen differed from BOP/VIP [3] in early dose intensity, use of infusional bleomycin, and use of BEP in the second treatment phase with higher dose etoposide than VIP [17].

CBOP/BEP results from previous studies [18], [19] suggested high activity with increased toxicity. However, case selection or improved management over time hinders historical comparisons. This randomised phase 2 trial (ISRCTN 53643604) evaluated CBOP/BEP and BEP at the same time.

## 2 Patients and methods

### 2.1 Patients

Applicable regulatory and ethics approvals and written informed consent were obtained. Eligible patients were ≥16 yr of age with a GCT of any extracranial primary site and International Germ Cell Cancer Collaborative Group (IGCCCG) poor-prognosis features (mediastinal primary, nonpulmonary visceral metastases, α-fetoprotein [AFP] >10 000 ng/ml, human chorionic gonadotropin [hCG] >50 000 IU/l, or lactase dehydrogenase >10 times the upper limits of normal). Diagnoses were based on histology or by elevated AFP and/or hCG in a patient with a testicular tumour, or unequivocally raised markers (AFP >1000 ng/ml or hCG >5000 IU/l) in men <45 yr of age without a testis tumour but with an otherwise appropriate clinical picture.

### 2.2 Study design

This open phase 2 multicentre trial randomised patients (1:1) to BEP or CBOP/BEP. Eligible patients not deemed fit enough to receive protocol chemotherapy could be stabilised with low-dose chemotherapy (normally cisplatin 20 mg/m^2^ or carboplatin area under the curve 3 and etoposide or vincristine for 2 d) prior to enrolment. Central randomisation through the trials unit used minimisation (with a random element) based on pre-protocol chemotherapy, primary tumour site, and centre.

### 2.3 Treatment

**Fig. 1:**
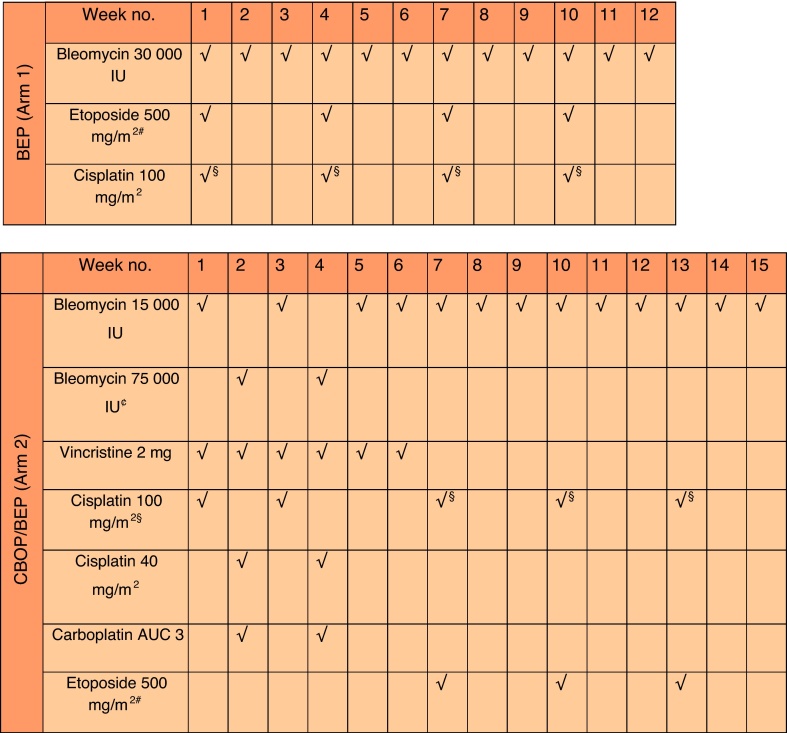
Chemotherapy regimens. AUC = area under the curve; BEP = bleomycin, etoposide, and cisplatin; CBOP/BEP = carboplatin, bleomycin, vincristine, cisplatin/bleomycin, etoposide, and cisplatin. ^#^ Etoposide to be given at 100 mg/m^2^ daily over 5 d. ^§^ Cisplatin to be given at 20 mg/m^2^ daily for 5 d. ^£^ Cisplatin to be given at 50 mg/m^2^ days 1 and 2 or 20 mg/m^2^ daily for 5 d (weeks 1 and 3). ^¢^ Bleomycin to be given at 15 000 IU by 24-h infusion daily over 5 d.

The control arm comprised four 3-weekly cycles of Indiana-style BEP, and the CBOP/BEP arm comprised six cycles over 15 wk (see Fig. 1 for doses).

Prophylactic granulocyte colony-stimulating factor (G-CSF) was mandated (in week 5 of CBOP/BEP and during each BEP cycle in both arms) from January 2008 following Independent Data Monitoring Committee advice.

Assessments prior to each cycle included physical examination/performance status; full blood count including AFP, hCG; chest x-ray; and renal function (including magnesium) with creatinine clearance performed during the first two cycles of CBOP.

Dose adjustments were made for myelosuppression according to day 1 counts and previous haematological toxicity; treatment cycles were omitted during CBOP until recovery. Neutropenic sepsis was treated immediately with broad-spectrum antibiotics. Dose modifications were also recommended for renal impairment, allergic reactions, and pulmonary or neurologic toxicity including permanent discontinuation of bleomycin in the case of lung toxicity.

Following treatment, patients were reviewed with clinical examination, chest X-ray, and tumour markers every 2 mo in year 1, every 3 mo in year 2, then every 6 mo until the end of year 5. Cross-sectional imaging was performed: 2–4 wk from the end of treatment; to follow residual disease every 6 mo until resolution (<1 cm), resected, or stable for 1 yr; 2 mo following surgical resection of tumour masses; and at the investigator's discretion. Surgical resection was advised for all nonresolving masses >1 cm. Management of disease progression was at the clinician's discretion.

### 2.4 Outcome measures

Response was evaluated as previously described, and a response category defined as listed below; the primary end point was the favourable response rate (FRR), the numerator comprising categories 1 and 2. *Complete response* was defined as normal AFP/hCG and either no clinical or radiologic evidence of disease, or complete resection of all residual masses; no viable tumour was found. *Partial response, marker negative* was defined as normal AFP/hCG and no surgery or partial resection only; no viable tumour was detected. *Treatment failure* was defined as the progressive rise of tumour markers at the end of treatment, an increase in tumour masses or appearance of new lesions not due to mature teratoma syndrome, or the presence of viable tumour in resected specimens. A subset of the latter group, referred to as *no evidence of disease (NED) after surgery*, was identified to include patients with normal markers and completely resected nontestis masses in which viable tumour was found.

Subsequently, recognising the testis as a sanctuary site, the Trial Management Group (blinded to study data) agreed that patients undergoing postchemotherapy orchidectomy with viable tumour found would be included in the *favourable response* category if all other features fitted this category. The prefix *late orchidectomy* identifies these patients in the results.

Secondary end points were progression-free survival (PFS), overall survival (OS), and toxicity. PFS was measured from randomisation to date of progression or death from any cause; patients with treatment failure, other than those in the NED subgroup, were counted as having an event on day 1 to avoid bias due to longer treatment duration with CBOP/BEP. Progressive disease was defined as rising tumour markers for >4 wk and/or increase in the size of lesions, or the appearance of new lesions (excluding growing mature teratoma).

### 2.5 Statistical considerations

A single-stage Fleming design was used, assuming an FRR for CBOP/BEP ≥80% would warrant further study and a rate <60% that historical data suggest with BEP [3] would not. With 44 patients randomised to CBOP/BEP, the trial had 90% power to exclude response rates ≤60% with α = 5% (one-sided) when the true response rate was ≥80%. Thus “success” for the primary analysis required the one-sided 95% confidence limit (equivalently, the lower limit of the 90% two-sided CI) to exclude rates <60%. An equal number were randomised to BEP to benchmark the FRR; the trial was not powered to compare arms definitively with respect to efficacy. Continuation to a phase 3 trial powered for PFS required success as defined earlier in the primary analysis, that the BEP FRR was within the anticipated range, and that trial recruitment rate was adequate.

Two sensitivity analyses were prespecified: (1) The *NED after surgery* group was included as favourable responders; (2) the *late orchidectomy partial response* group was excluded from the favourable response category. FRRs were also assessed according to receipt of pre-protocol stabilising chemotherapy (planned subgroup analysis) and according to histologic diagnosis (exploratory analysis). A per protocol population was defined a priori to include eligible patients receiving one cycle or more of protocol chemotherapy; however, all patients met these criteria. Preplanned time-to-event analyses included Kaplan-Meier curves for PFS and OS with treatment hazard ratios (HRs) derived from Cox regression models.

## 3 Results

**Fig. 2:**
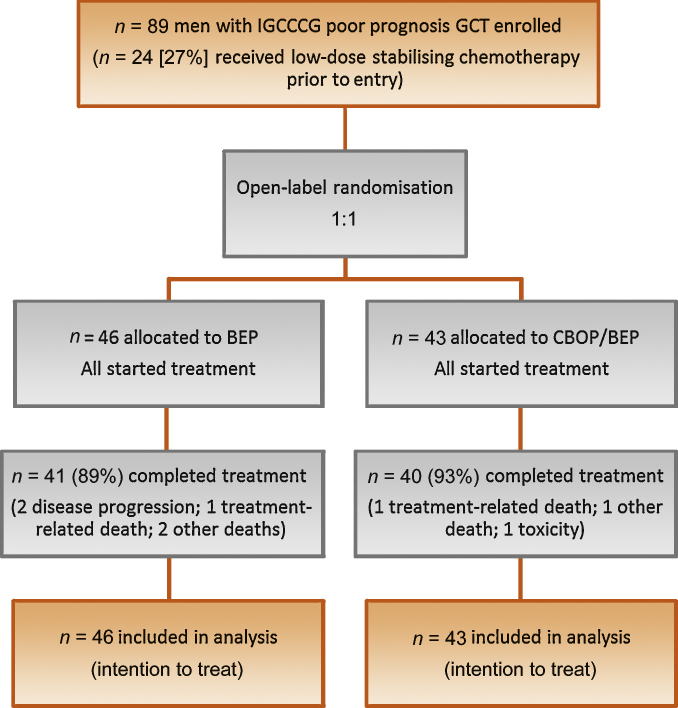
Trial profile. BEP = bleomycin, etoposide, and cisplatin; CBOP/BEP = carboplatin, bleomycin, vincristine, cisplatin/bleomycin, etoposide, and cisplatin; GCT = germ cell tumour; IGCCCG = International Germ Cell Cancer Collaborative Group.

A total of 89 patients were randomised from 16 UK centres, 46 to BEP and 43 to CBOP/BEP (Fig. 2). Baseline characteristics were well balanced (Table 1). Mean age was 30 yr (range: 16–68); 18 (20%) had mediastinal primary tumours. Overall, 53 patients (60%) had diagnosis confirmed histologically and 36 (40%) based on markers/clinical picture. Twenty-four patients (27%) had pre-protocol low-dose chemotherapy for stabilisation.

**Table 1:** Baseline characteristics

	BEP	CBOP/BEP	Overall
No. of patients	46	43	89
Age, yr			
Mean (SD)	31.3 (10.7)	28.5 (8.8)	29.9 (9.9)
Site of primary tumour, *n* (%)			
Testis	34 (72)	32 (74)	66 (74)
Mediastinum	9 (20)	9 (21)	18 (20)
Retroperitoneum	2 (4)	2 (5)	4 (4)
Other*	1 (4)	0 (0)	1 (1)
IGCCCG risk factors, *n* (%)			
Raised markers† only	14 (30)	10 (23)	24 (27)
Mediastinal primary tumour only	7 (15)	4 (9)	11 (12)
NPVM only	9 (20)	10 (23)	19 (21)
Raised markers and mediastinal primary	2 (4)	3 (7)	5 (6)
Raised markers and NPVM	14 (30)	14 (33)	28 (31)
Mediastinal primary and NPVM	0 (0)	2 (5)	2 (2)
Stabilising chemotherapy prior to protocol treatment, *n* (%)	13 (28)	11 (26)	24 (27)

BEP = bleomycin, etoposide, and cisplatin; CBOP = carboplatin, bleomycin, vincristine, cisplatin; IGCCCG = International Germ Cell Cancer Collaborative Group; NPVM = nonpulmonary visceral metastases; SD = standard deviation.

* Difficult to determine between testis and retroperitoneum.

† α-Fetoprotein > 10 000 ng/ml, human chorionic gonadotropin > 50 000 IU/l, or lactate hydrogenase > 10 times upper limit of normal.

### 3.1 Treatment

Treatment completion rates for BEP and CBOP/BEP, respectively, were 41 of 46 (89%) and 40 of 43 (93%); reasons for early stopping were disease progression (2 BEP, 0 CBOP/BEP), toxicity-related death (1 BEP, 1 CBOP/BEP), other early death (2 BEP, 1 CBOP/BEP), and toxicity (0 BEP, 1 CBOP/BEP). Dose modifications or omissions occurred in 34 CBOP/BEP patients (79%) versus 16 (35%) on BEP. For BEP, these were most commonly omissions of bleomycin in later cycles. For CBOP/BEP, omissions were most common in cycle 3 (weeks 5 and 6): 15 patients (36%) who received three or more cycles had one or more drug doses omitted from cycle 3 including four who missed this cycle altogether (as mandated for significant myelosuppression) but went on to complete treatment. In addition, three CBOP/BEP patients were given bleomycin at 30 000 IU rather than 15 000 IU during the BEP cycles in error (in one case, all nine weekly doses; in two cases, seven doses with the remaining two omitted).

### 3.2 Toxicity and deaths during treatment

With CBOP/BEP, 95% had Common Terminology Criteria for Adverse Events v.3 grade ≥3 symptoms during treatment versus 63% of BEP patients (Table 2), largely due to haematologic toxicity, particularly neutropenia that affected 25 (54%) and 36 (84%) patients, respectively; 7 (15%) and 13 (30%), respectively, had neutropenic fever. Grade 3–4 thrombocytopenia occurred in 8 (18%) BEP and 23 (54%) CBOP/BEP patients.

**Table 2:** Grade 3 or 4 worst toxicity during chemotherapy

Toxicity	CTCAE grade*	BEP, *n* (%)	CBOP/BEP, *n* (%)
Thrombocytopenia	3	4 (9)	6 (14)
	4	4 (9)	17 (40)
Neutropenia	3	14 (30)	8 (19)
	4	11 (24)	28 (65)
Fever with grade 3 or 4 neutropenia	3	0 (0)	7 (16)
	4	0 (0)	0 (0)
Other haematologic symptoms	3	9 (20)	20 (47)
(not specified)	4	0 (0)	7 (16)
Anorexia	3	3 (7)	3 (7)
	4	0 (0)	0 (0)
Constipation	3	0 (0)	1 (2)
	4	0 (0)	0 (0)
Diarrhoea	3	2 (4)	4 (9)
	4	0 (0)	1 (2)
Nausea	3	2 (4)	8 (19)
	4	0 (0)	1 (2)
Vomiting	3	2 (4)	4 (9)
	4	0 (0)	1 (2)
Fever	3	0 (0)	8 (19)
	4	0 (0)	1 (2)
Sensory neuropathy	3	0 (0)	1 (2)
	4	0 (0)	0 (0)
Dermatologic	3	1 (2)	0 (0)
	4	0 (0)	0 (0)
Fatigue	3	2 (4)	9 (21)
	4	0 (0)	0 (0)
Auditory	3	0 (0)	1 (2)
	4	0 (0)	0 (0)
Vascular	3	0 (0)	3 (7)
	4	0 (0)	0 (0)
Cardiovascular	3	1 (2)	1 (2)
	4	1 (2)	1 (2)
Pulmonary	3	2 (4)	3 (7)
	4	1 (2)	1 (2)
Renal	3	0 (0)	0 (0)
	4	0 (0)	0 (0)
Pain	3	3 (7)	6 (14)
	4	0 (0)	0 (0)
Other toxicity	3	3 (7)	14 (33)
	4	0 (0)	3† (7)

BEP = bleomycin, etoposide, and cisplatin; CBOP = carboplatin, bleomycin, vincristine, cisplatin; CTCAE = Common Terminology Criteria for Adverse Events v.3.

* As reported at the time of scheduled assessment. Any subsequent deaths thought to be related to treatment are reported in the text.

† Pulmonary embolism; leukocytes; line infection.

Distinguishing between symptoms of severe disease, toxicity, and intercurrent illness or infection made it difficult to classify cause of death in several cases. Two on-treatment deaths in the CBOP/BEP arm (one in a patient who had received a bleomycin overdose) and one BEP death 3 mo after treatment were thought likely to be a result of lung damage associated with bleomycin. Bleomycin toxicity was also a possible contributory factor in three further deaths (one BEP, two CBOP/BEP) from infective respiratory conditions (postoperative adult respiratory distress syndrome [ARDS] [one], pneumonia [two]) that occurred 2–3 mo after completion of protocol chemotherapy.

A further two non-GCT deaths on each arm occurred during treatment. In the BEP arm these were due to haemorrhage of brain metastases when heparinised (a complication of neutropenic sepsis management) and a combination of severe disease, toxicity, and pneumonia after the first week of BEP. In the CBOP/BEP arm they were due to ARDS and septicaemia (a complication of neutropenic sepsis that developed in the first week of treatment) and to multiorgan failure associated with sepsis in a patient who was not neutropenic.

The policy change mandating G-CSF prophylaxis led to 98% (previously 40%) of BEP patients and 76% (previously 47%) of CBOP/BEP patients receiving G-CSF in the required cycles. However it did not have a substantial impact on the incidence or grade of neutropenia or febrile neutropenia (29% in CBOP/BEP before and after the protocol amendment), although an impact on duration of symptoms (not assessable) cannot be excluded.

### 3.3 Efficacy

Response was evaluated in all 89 patients (Table 3). FRRs were 60.9% (90% CI, 47.7–73.0) for the BEP arm and 74.4% (90% CI, 61.2–84.9) for the CBOP/BEP arm. Results from the sensitivity analyses (Table 3) were broadly consistent.

**Table 3:** Response to treatment

Response to treatment	BEP	CBOP/BEP	Overall
No. of patients	46	43	89
Favourable: primary analysis, *n* (%)			
Complete response	4 (9)	12 (28)	16 (18)
Partial response: negative markers	23 (50)	18 (42)	41 (46)
Late orchidectomy and partial response*	1 (2)	2 (5)	3 (3)
Nonfavourable, *n* (%)			
No evidence of disease after surgery**	2 (4)	0 (0)	2 (2)
Treatment failure	11 (24)	6 (14)	17 (19)
Mixed response	0 (0)	1 (2)	1 (1)
Death due to germ cell tumour	2 (4)	0 (0)	2 (2)
Death due to toxicity	1 (2)	3 (7)	4 (4)
Death due to other reason	2 (4)	1 (2)	3 (3)
Primary analysis: favourable response, *n* (%)	28 (60.9)	32 (74.4)	60 (67.4)
90% confidence interval	47.7–73.0	61.2–84.9	58.3–75.6
First sensitivity analysis			
Favourable response, *n* (%)	30 (65.2)	32 (74.4)	62 (69.7)
90% confidence interval	51.1–76.8	61.2–84.9	60.7–77.6%
Second sensitivity analysis			
Favourable response, *n* (%)	27 (58.7)	30 (69.8)	57 (64.0)
90% confidence interval	45.5–71.0	56.3–81.1	54.9–72.5
Subgroup†: patients *not* given preprotocol chemotherapy			
No. of patients	33	32	65
Favourable response, *n* (%)	22 (66.7)	24 (75.0)	46 (70.8)
90% confidence interval	50.9–80.1	59.4–86.9	60.1–79.9
Subgroup†: patients given pre-protocol chemotherapy			
No. of patients	13	11	24
Favourable response, *n* (%)	6 (46.2)	8 (72.7)	14 (58.3)
90% confidence interval	22.4–71.3	43.6–92.1	39.7–75.4
Subgroup†: patients *with* a histologic diagnosis at enrolment			
No. of patients	29	24	53
Favourable response, *n* (%)	18 (62.1)	16 (66.7)	34 (64.2)
90% confidence interval	45.1–77.1	47.9–82.2	52.0–75.1
Subgroup†: patients *without* a histologic diagnosis at enrolment			
No. of patients	17	19	36
Favourable response, *n* (%)	10 (58.8)	16 (84.2)	26 (72.2)
90% confidence interval	36.4–78.8	64.1–95.6	57.4–84.1

BEP = bleomycin, etoposide, and cisplatin; CBOP = carboplatin, bleomycin, vincristine, cisplatin.

* Normal markers, residual nontestis mass remains, postchemotherapy orchidectomy (viable tumour)

** Normal markers, complete resection of residual nontestis mass, viable tumour found.

† The subgroup analysis according to use of pre-protocol chemotherapy was planned; analysis according to histologic diagnosis is exploratory. Of 36 patients without histology, 10 also had preprotocol chemotherapy.

The FRR was 58.3% (39.7–75.4%) in patients having pre-protocol stabilising chemotherapy and 70.8% (60.1–79.9%) in those that did not, and it was 64.2% (52.0–75.1%) in those with a histologic diagnosis compared with 72.2% (57.4–75.1%) in those without. The difference between arms appeared more marked in both those receiving pre-protocol chemotherapy and those without a histologic diagnosis (Table 3).

**Fig. 3:**
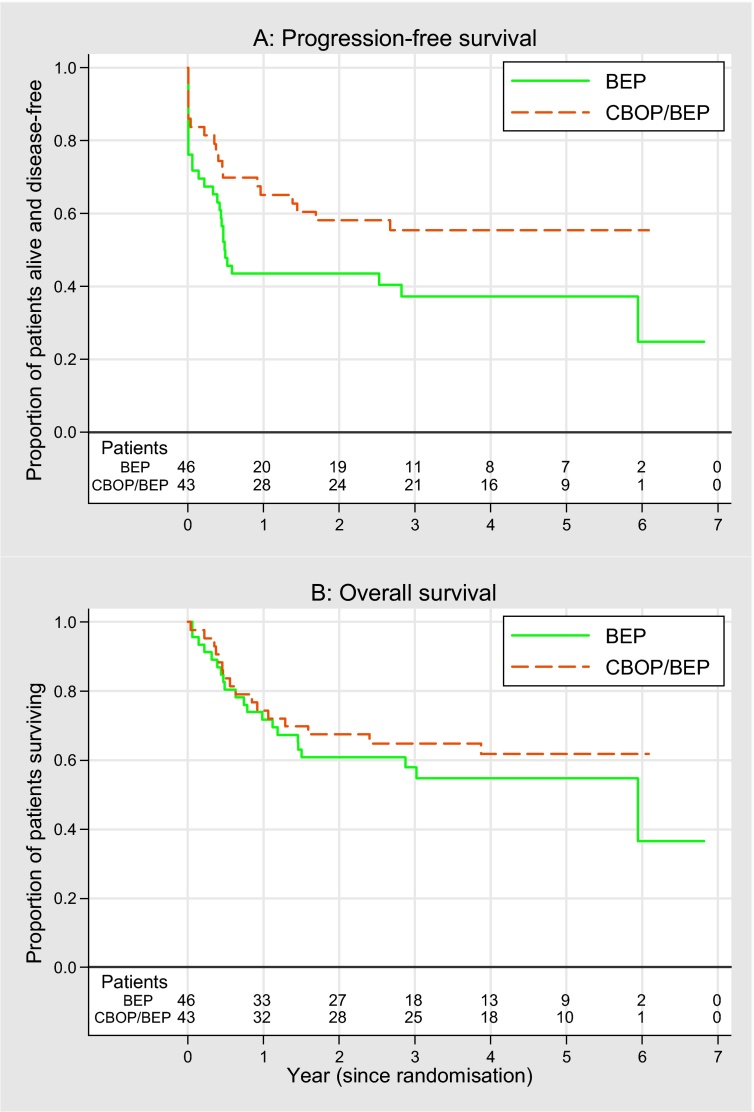
Kaplan-Meier plots for (A) progression-free survival and (B) overall survival. BEP = bleomycin, etoposide, and cisplatin; CBOP/BEP = carboplatin, bleomycin, vincristine, cisplatin/bleomycin, etoposide, and cisplatin.

Follow-up data were updated in December 2012. Median follow-up was 58 mo, with a minimum 18-mo follow-up for progression-free patients. There were 48 PFS events and 37 deaths (Fig. 3). The 1-yr PFS rates were 43% (95% CI, 29–57) for BEP and 65% (95% CI, 49–77%) for CBOP/BEP; HR: 0.59 (95% CI, 0.33–1.06). Two-year survival rates were 61% for BEP and 67% for CBOP/BEP (HR: 0.78 [95% CI, 0.41–1.50]).

There were 14 non-GCT deaths (6 BEP, 8 CBOP/BEP) in total. In addition to the 10 deaths that occurred during and/or were considered related to protocol treatment, described previously, 4 later deaths occurred that were not due to GCT: two (one in each arm) due to toxicity from second-line treatment; one thought likely due to recurrent teratoma, but a second nonhaematologic malignancy could not be ruled out (CBOP/BEP); and one due to primary lung cancer (BEP).

## 4 Discussion

The primary efficacy end point was met, with BEP FRRs approximately as anticipated (61%); the FRR with CBOP/BEP was 74%, with the 90% CI excluding rates <60%. This was supported by encouraging PFS and OS data, particularly in patients with aggressive disease needing stabilising chemotherapy; however, acute toxicity with CBOP/BEP was high. Accrual was slower than anticipated; hence the criteria for proceeding immediately to phase 3 were not met, and it is clear that an adequately powered phase 3 trial would require international collaboration.

The two previous nonrandomised CBOP/BEP studies also suggested high activity with increased toxicity. The first [18], in 54 IGCCCG poor-prognosis patients, showed 3-yr relapse-free survival of 83.2% (95% CI, 68.8–91.3) and 3-yr survival of 91.5% (95% CI, 78.6–96.8). In European Organisation for Research and Treatment of Cancer (EORTC) 30948 [19], 29 of 66 eligible patients had poor prognosis. After a median 40-mo follow-up, 1-yr PFS in the poor-prognosis group was 81.8% (95% CI, 72.5–91.1). Two-year survival was 84.5% (95% CI, 75.6–93.3). In both studies, the major toxicities were haematologic. There were three treatment-related deaths in the first study but none in the second.

A systematic review (including searches of Medline, American Society of Clinical Oncology annual meeting abstracts [2007–2013], and reference lists from related reviews [20], [21], [22]) identified 12 randomised trials of novel treatments versus BEP in intermediate- or poor-prognosis patients (Table 4). Even the largest of these was only powered to detect an absolute PFS benefit of 15% [3]; several failed to recruit the targeted sample size. With the exception of the Genito-Urinary Group of the French Federation of Cancer Centres (GETUG) 13 trial [23], which randomised a subset of poor-risk patients showing inadequate marker decline after 1 × BEP to continue BEP or switch to a dose-dense regimen, none show clear superiority over BEP.

**Table 4:** Phase 2 and 3 randomised trials of alternative treatments to standard BEP for intermediate and poor prognosis germ cell tumours

Study	Accrual years	Prognostic group (classification criteria)	Test regimen	*N* (*n* on test)	Event-free survival at 2 yr^#^ (HR < 1.0 favours test regimen)			Overall survival at 5 yr^#^ (HR < 1.0 favours test regimen)		
					BEP	Test	HR (95% CI)	BEP	Test	HR (95% CI)
De Wit et al. [30]	1983–1987	Poor (EORTC)	PVB/BEP	234 (116)	≥80%	≥80%	–	∼79%	∼81%	∼0.89
Nichols et al. [31]	1984–1989	Poor (Indiana)	BEP200	153 (76)	61%*	63%*	∼0.93	∼69%	∼63%	∼1.25
De Wit et al. [32]	1987–1990	Intermediate (EORTC)	VIP	87 (46)	∼85%	∼85%	0.83 (0.3–2.28)	∼95%**	∼98%**	∼0.39
Nichols et al. [4]	1987–1992	Poor (Indiana)	VIP	286 (145)	60%	64%	∼0.87	∼66%	∼68%	∼0.93
Kaye et al. [3]	1990–1994	Poor (MRC/EORTC) (63% IGCCCG poor)	BOP/VIP-B	380 (190) 216 (108)	∼57% –	∼49% –	1.28 (0.95–1.72) 1.04 (0.73–1.50)	∼73% –	∼70% –	1.30 (0.88–1.92) 0.99 (0.62–1.58)
Culine et al. [24]	1994–2000	All Intermediate (IGCCCG) Poor (IGCCCG)	CISCA/VB	185 (94) 61 (30) 115 (57)	∼51% ∼70% ∼37%	∼37% ∼56% ∼24%	1.32 (0.90–1.92) ∼1.44	69% 88% 69%	58% 82% 44%	1.37 (0.85–2.17) ∼2.21
Motzer et al. [6]	1994–2003	All Intermediate (IGCCCG) Poor (IGCCCG)	BEP × 2 HD CEC × 2	219 (108) 45 (21) 174 (87)	∼47% – ∼45%	∼50% – ∼46%	∼0.92 ∼0.97	71% 83% 69% at 2 yr	71% 85% 67% at 2 yr	∼1.0 – ∼1.08
Di Nicola et al. [25]	1996–2007	Poor (IGCCCG)	BEP × 2 HD-carboplatin	89 (43)	59%	56%	∼1.10	67%	61%	∼1.23
Daugaard et al. [7] (EORTC 30974)	1999–2007	Poor (IGCCCG)	VIP × 1 HD VIP × 3	131 (65)	45%	58%	0.62 (0.38–1.02)	∼60%	∼67%	∼0.78
De Wit et al. [26] (EORTC 30983)	1998–2009	Intermediate (IGCCCG)	T-BEP	337 (168)	∼75%	∼82%	0.73 (0.47–1.13)	∼88%	∼90%	0.89 (0.46–1.74)
Huddart (NCRI TE23)	2005–2009	Poor (IGCCCG)	CBOP/BEP	89 (43)	43%	58%	0.59 (0.33–1.06)	58% at 3 yr	65% at 3 yr	0.78 (0.41–1.50)
Fizazi et al. [23] (GETUG 13)		Poor (IGCCCG) with unfavourable TMD	T-BEP and Ox × 2, PIB × 2	203 (105)	48% at 3 yr	59% at 3 yr	∼0.72	65% at 3 yr	73% at 3 yr	∼0.73

BEP = bleomycin, etoposide, and cisplatin; BOP/VIP-B = bleomycin, vincristine, cisplatin/etoposide, ifosfamide, cisplatin, and bleomycin; CBOP/BEP = carboplatin, bleomycin, vincristine, cisplatin/bleomycin, etoposide, and cisplatin; CI = confidence interval; CEC = carboplatin, etoposide, and cyclophosphamide; CISCA-VB = cisplatin, doxorubicin and cyclophosphamide alternated with vinblastine and bleomycin; EORTC = European Organisation for Research and Treatment of Cancer; GETUG = Genito-Urinary Group of the French Federation of Cancer Centers; HD = high dose; HR = hazard ratio; IGCCCG = International Germ Cell Cancer Collaborative Group; MRC = Medical Research Council; NCRI = National Cancer Research Institute; NED = no evidence of disease; OX = oxaliplatin; PIB = cisplatin, ifosfamide and bleomycin; PVB = cisplatin, vinblastine, bleomycin; T-BEP = paclitaxel and bleomycin, etoposide, and cisplatin; TMD = tumor marker decline; VIP = cisplatin, etoposide, and ifosfamide.

# In these columns “∼” indicates event-free rates estimated from Kaplan-Meier curves and HRs estimated as ln(p2)/ln(p1) where p2 = event-free rate on test, p1 = event-free rate on BEP. * Event-free survival = percentage continuously NED with median follow-up 2 yr.

** Overall survival = crude survival rate, median follow-up 7.7 yr.

Results of high-dose chemotherapy trials [6], [7], [24], [25] are notably divergent and were most favourable in EORTC 30974 that closed early due to poor accrual. CBOP/BEP compares well, demonstrating the greatest estimated relative PFS benefit over BEP. Both the BEP results and relative benefit are remarkably similar to those attained in EORTC 30974 [7] despite what appears to be a higher treatment-related death rate in the present study. Overall grade 3/4 toxicity rates are not presented for EORTC 30974, but grade 4 neutropenia rates were higher arm for arm than in TE23 (47% vs 24% for BEP; 82% high dose vs 65% CBOP/BEP). Rates of grade 4 leukopenia (11%, 20%) and thrombocytopenia (3%, 12%) across the BEP arms of EORTC trials in intermediate [26] and poor-prognosis disease [7], respectively, are suggestive of increased toxicity associated with more advanced disease even on standard therapy. Nevertheless, these more intensive schedules do carry the risk of increased toxicity, so the risk–benefit ratio may be debatable. Any such debate does need to consider the impact of salvage treatment on cumulative toxicity burden in less intensively treated patients who relapse.

Restricting more intensive treatment to those most in need is desirable. Use of dynamic markers to identify patients who are not responding sufficiently well to standard therapy, as in GETUG 13, is a promising strategy, although the optimal time point to assess rate of marker decline and the method of intensification is still debatable. A further potential strategy for which there are limited data at present [27], [28], [29] is dose density, giving BEP every 2 rather than every 3 wk, with G-CSF support.

## 5 Conclusions

Improved treatments for poor-prognosis disease that increase cure rate by first-line therapy are needed. At present, there is no accepted alternative to BEP, and to challenge its status as standard therapy requires an adequately powered phase 3 trial. This in turn will need international agreement. Because CBOP/BEP met its preset activity goals and attained PFS rates equivalent or better than other approaches to poor-risk disease, it merits consideration in the development of any such trial.

  ***Previous presentation:*** Some of the results included in the manuscript were presented at the ASCO Annual Meeting 2011: Huddart RA, Gabe R, Cafferty F, et al. A randomized phase II trial of intensive induction chemotherapy (CBOP/BEP) and standard BEP in poor prognosis germ cell tumors (MRC TE23, CRUK 05/014, ISRCTN53643604) [abstract 4508]. J Clin Oncol 2011;29:15s(Suppl).  ***Author contributions:*** Robert A. Huddart had full access to all the data in the study and takes responsibility for the integrity of the data and the accuracy of the data analysis.  

*Study concept and design:* Huddart, Stenning.

*Acquisition of data:* Huddart, White, Shamash, Cullen, Pollock.

*Analysis and interpretation of data:* Gabe, Cafferty, Stenning, Huddart.

*Drafting of the manuscript:* Huddart, Gabe, Cafferty, Stenning.

*Critical revision of the manuscript for important intellectual content:* Pollock, White, Shamash, Cullen.

*Statistical analysis:* Gabe, Cafferty, Stenning.

*Obtaining funding:* Huddart, Stenning.

*Administrative, technical, or material support:* Pollock.

*Supervision:* Stenning.

*Other* (specify): None.  

***Financial disclosures:*** Robert A. Huddart certifies that all conflicts of interest, including specific financial interests and relationships and affiliations relevant to the subject matter or materials discussed in the manuscript (eg, employment/affiliation, grants or funding, consultancies, honoraria, stock ownership or options, expert testimony, royalties, or patents filed, received, or pending), are the following: None.  

***Funding/Support and role of the sponsor:*** This study was funded by Cancer Research UK (CRUK/05/014) with additional support from the Medical Research Council through the Clinical Trials Unit.  

***Acknowledgement statement:*** We thank all the participants and their families and acknowledge support to investigators (see Supplement 1) from the National Institute for Health Research through the UK National Cancer Research Network.
