# Supplementary material for: A Randomised Phase 2 Trial of Intensive Induction Chemotherapy (CBOP/BEP) and Standard BEP in Poor-prognosis Germ Cell Tumours (MRC TE23, CRUK 05/014, ISRCTN 53643604)
Source: Eur Urol. 2015 Mar;67(3):534–43. doi: 10.1016/j.eururo.2014.06.034 (PMC4410298; doi:10.1016/j.eururo.2014.06.034)
Supplement: Supplementary file 1 [file mmc1.doc]

**Supplement 1 – Acknowledgements for trial organisation, oversight, and participation**

The trial was coordinated by the Medical Research Council Clinical Trials Unit: statisticians Fay Cafferty, Rhian Gabe, Patrick Fogarty and Sally Stenning; trial managers Phillip Pollock, Ben Spittle, and Lisa McDonald; and data managers Hassan Khan, James Pickering, and Montse Wells.

In addition to the authors and staff at the Clinical Trials Unit, the Trial Management Group included John Chester (Velindre Hospital, Cardiff), Annelies Gillesen (University College Hospital), Ben Mead (retired, Southampton General Hospital), Sue Rodwell (St. James University Hospital, Leeds), and Zoe Whittington (Parkside Oncology Clinic, London).

The Independent Data Monitoring Committee was chaired by Judith Bliss (Institute of Cancer Research, Sutton) with Hans Schmoll (University Clinic, Halle, Germany) and Graham Read (The Lancashire and Lakeland Cancer Centre Royal Preston Hospital/Lancashire Teaching Hospitals NHS Trust). The Independent Trial Steering Committee was chaired by David Guthrie (Derby) with John Scholefield (University Hospital, Nottingham) and Richard Cowan (Christie Hospital NHS Trust, Manchester).

The following investigators and local research teams participated in the trial:

| Investigator | Site | Patients enrolled |
| --- | --- | --- |
| J. White | Beatson West of Scotland Cancer Centre (Glasgow) | 15 |
| R.A. Huddart | Royal Marsden Hospital (Sutton) | 9 |
| J. Shamash | St. Bartholomew’s Hospital (London) | 9 |
| M.P. Sokal, I. Hennig | Nottingham City Hospital (Nottingham) | 8 |
| M.H. Cullen, S.A. Hussain | Queen Elizabeth Hospital (Birmingham) | 8 |
| G.M. Mead, P.D. Simmonds | Southampton General Hospital (Southampton) | 7 |
| R. Brown, P. Rogers | Royal Berkshire Hospital (Reading) | 6 |
| R. Welch, M. Leahy | Christie Hospital (Manchester) | 5 |
| J. Braybrooke,R. Jones | Bristol Haematology & Oncology Centre (Bristol) | 5 |
| D. Stark, J. Joffe | St. James University Hospital (Leeds) | 4 |
| R. Owen | Gloucestershire Oncology Centre (Cheltenham) | 4 |
| S. Harland | University College London Hospital (London) | 4 |
| S. Nicholson | Leicester Royal Infirmary (Leicester) | 2 |
| A. Hong | Royal Devon and Exeter Hospital (Exeter) | 1 |
| M. Williams | Addenbrookes NHS Trust (Cambridge) | 1 |
| A. Birtle, W. Appel | Royal Preston Hospital (Preston) | 1 |
| P. Clarke | Clatterbridge Centre for Oncology (Wirral) | 0 |
| C. Humber | University Hospitals Coventry & Warwick (Coventry) | 0 |
| J. Barber | Velindre Hospital (Cardiff) | 0 |
